# Self-blame-selective hyper-connectivity between anterior temporal and subgenual cortices predicts prognosis in major depressive disorder

**DOI:** 10.1016/j.nicl.2023.103453

**Published:** 2023-06-16

**Authors:** Diede Fennema, Gareth J. Barker, Owen O'Daly, Suqian Duan, Ewan Carr, Kimberley Goldsmith, Allan H. Young, Jorge Moll, Roland Zahn

**Affiliations:** a Centre of Affective Disorders, Institute of Psychiatry, Psychology & Neuroscience, Centre for Affective Disorders, King’s College London, London, UK; b Department of Neuroimaging, Institute of Psychiatry, Psychology & Neuroscience, King’s College London, London, UK; c Department of Biostatics and Health Informatics, Institute of Psychiatry, Psychology & Neuroscience, King’s College London, London, UK; d Cognitive and Behavioural Neuroscience Unit, D’Or Institute for Research and Education (IDOR), Rio de Janeiro, Brazil; e National Service for Affective Disorders, South London and Maudsley NHS Foundation Trust, London, UK

**Keywords:** fMRI, Self-blame, Depression, Biomarker, Prognosis

## Abstract

**Background:**

Self-blame-related fMRI measures were shown to predict subsequent recurrence in remitted major depressive disorder (MDD). Their role in current MDD, however, is unknown. We hypothesised that these neural signatures reflect a highly recurrent but remitting course of MDD and therefore predict favourable outcomes over a four-month follow-up period in current MDD.

**Methods:**

Forty-five participants with current MDD and non-responders to at least two serotonergic antidepressants, were encouraged to optimise their medication and followed up after receiving four months of primary care treatment-as-usual. Prior to their medication review, participants completed an fMRI paradigm in which they viewed self- and other-blame emotion-evoking statements. Thirty-nine participants met pre-defined fMRI data minimum quality thresholds. Psychophysiological interaction analysis was used to determine baseline connectivity of the right superior anterior temporal lobe (RSATL), with an *a priori* BA25 region-of-interest for self-blaming vs other-blaming emotions, using Quick Inventory of Depressive Symptomatology (16-item) percentage change as a covariate.

**Results:**

We corroborated our pre-registered hypothesis that a favourable clinical outcome was associated with higher self-blame-selective RSATL-BA25 connectivity (Family-Wise Error-corrected *p* <.05 over the *a priori* BA25 region-of-interest; *r_s_*(34) = −0.47, *p* =.005). This generalised to the sample including participants with suboptimal fMRI quality (*r_s_*(39) = −0.32, *p* =.05).

**Conclusions:**

This study shows that neural signatures of overgeneralised self-blame are relevant for prognostic stratification of current treatment-resistant MDD. Future studies need to confirm whether this neural signature indeed represents a trait-like feature of a fully remitting subtype of MDD, or whether it is also modulated by depressive state and related to treatment effects.

## Highlights

• Self-blame-related fMRI measures were shown to predict recurrence risk of depression.
• Their prognostic value in treatment-resistant forms of depression was examined.
• Neural signatures of self-blame are relevant for prognostic stratification.

## 1 Introduction

Approximately only one in two patients with major depressive disorder (MDD) respond to their antidepressant treatment (Rush et al., 2006, Souery et al., 2007, Thomas et al., 2013), while more than a third fail to achieve remission even after several rounds of treatment (Al-Harbi, 2012, Gaynes et al., 2009, Strawbridge et al., 2022). If we were able to identify markers of poor prognosis in these patients, we could develop personalised treatment algorithms and pathways based on prognostic markers. Identifying such markers requires a deeper understanding of the pathophysiology and potential subtypes of MDD. For instance, epidemiological studies show that MDD follows distinct courses: 50% of patients have one episode, 35% have a fully remitting but recurring form, and 15% have a chronic course (Eaton et al., 2008). Guilt has classically been associated with familial and melancholic forms of depression (Leckman et al., 1984), which were thought to exhibit more severe episodes, but a better recovery compared with anxious (“neurotic”) and more chronic forms of depression (Gili et al., 2012). The functional anatomy of self-blame could therefore provide important insights into pathophysiological differences between prognostic subtypes of depression.

MDD is often described as being associated with negative cognitive biases (Disner et al., 2011), including increased negative self-referential thinking, i.e. a tendency to strongly introspect and reflect upon oneself (Grimm et al., 2009, Lemogne et al., 2009). Patients with remitted and current MDD exhibited lower levels of blame-related emotions towards others relative to themselves, i.e. showed a self-blaming emotional bias (Duan et al., 2023, Green et al., 2013, Zahn et al., 2015), rather than an overall increase in negative emotions (Watson et al., 1988). Psychopathological studies have highlighted the importance of self-blame-related emotions as symptoms of MDD, such as overgeneralised guilt and self-disgust (Green et al., 2013, Zahn et al., 2015, Zahn et al., 2015) and associated feelings of worthlessness (Harrison et al., 2022). These feelings do not only entail negative self-reference, but also require a concern about other people and the consequences of one’s actions for others (Taihara and Malik, 2016, Tangney et al., 2007), as well as an overgeneralised feeling of control for other people’s wrongdoings (Duan et al., 2021, O'Connor et al., 2002). Interestingly, proneness to experience self-blaming emotions remains detectable upon remission of symptoms, which suggests a role in MDD vulnerability (Ghatavi et al., 2002, Green et al., 2013, Zahn et al., 2015).

The subgenual frontal region and its connectivity with the right superior anterior temporal lobe (RSATL) have been proposed to play a key role in overgeneralised self-blaming emotions, characteristic of MDD (Zahn et al., 2020) and predicted recurrence risk (Lythe et al., 2015). It is unclear, however, what role self-blame-related neural signatures play in current and more chronic, treatment-resistant forms of MDD, and whether they have prognostic value in a primary care setting. The subgenual frontal region has been consistently associated with treatment response across imaging modalities (Dichter et al., 2015, Dunlop et al., 2019, Keedwell et al., 2010, Mayberg et al., 1997), but its functional contribution remains elusive.

In this pre-registered study (NCT04342299), we sought to determine whether self-blame-related fMRI measures are prospectively associated with clinical outcomes after four months of primary care treatment-as-usual. Of particular interest was the neural signature of connectivity between the posterior subgenual cortex (BA25) with the RSATL, as its self-blame-selective increases, i.e. those effects that were shown relative to other-blame, were associated with subsequent recurrence risk in fully remitting forms of MDD (Lythe et al., 2015). We speculated that this neural signature reflects a highly recurrent but fully remitting course of MDD and that it represents a marker of favourable prognosis for symptom remission as the Lythe et al. (2015) study was carried out in people with full remission and, thus, was likely to have been biased towards patients with an overall lower probability of a chronic course. Although bipolar disorders were excluded from our previous and the current study, highly recurrent MDD is associated with risk of bipolar disorder and is considered a bipolar characteristic (Angst et al., 2011). Bipolar features more generally were associated with a favourable short-term prognosis for symptomatic MDD and higher risk of recurrence (Vergunst et al., 2013). Therefore, we hypothesised (Hypothesis 1) that self-blame-selective RSATL-subgenual cortex (BA25) hyper-connectivity would be associated with favourable clinical outcomes after receiving four months of primary care treatment-as-usual.

In addition, Lythe et al. (2015) noted that hyper-connectivity of the RSATL with the ventral putamen / claustrum and with the temporoparietal junction was characteristic of participants with recurring MDD compared with participants who remained in stable remission. While the RSATL-temporoparietal junction hyper-connectivity did not survive a more stringent analysis applying an uncorrected cluster-forming threshold of *p* = 0.001 (Fennema et al., 2021), additional unpublished data collected by Lythe et al. (2015) revealed that people with a history of achieving remission using alternatives to serotonergic medications, e.g. psychotherapy or spontaneous remission, showed stronger RSATL hyper-connectivity with the right ventral striatum compared with serotonergic responders. The groups were well-matched on demographic and clinical variables, which suggests that the lower RSATL-ventral striatum connectivity cannot be explained by other confounders and might represent a genuine marker for serotonergic responders. This might be particularly relevant in the context of response to treatment in a primary care setting, which mostly relies on serotonergic medication. Thus, we further hypothesised (Hypothesis 2) that self-blame-selective RSATL-ventral striatum hyper-connectivity would be associated with poorer clinical outcomes after receiving four months of primary care treatment-as-usual.

Lastly, we hypothesised (Hypothesis 3) that patients with anxious distress would have a lack of self-blame-selective RSATL-BA25 hyper-connectivity. This was based on a recent randomised controlled neurofeedback trial investigating the potential of reinforcing decreasing RSATL-posterior subgenual cortex (BA25) connectivity for self-blaming emotions in current MDD, which was compared to a solely psychological intervention of self-blame-reducing strategies without neurofeedback (Jaeckle et al., 2021). Notably, patients with anxious MDD, commonly encountered in treatment-resistant and chronic MDD populations and which is associated with a poor prognosis (Dold et al., 2017, Domschke et al., 2010, Fava et al., 2008, Gaspersz et al., 2017), did not benefit from neurofeedback (Jaeckle et al., 2021). It is plausible that the neurofeedback interfered with the beneficial effects of psychological strategies in anxious MDD, which suggests that self-blame-selective RSATL-BA25 hyper-connectivity is pathophysiologically irrelevant to depressive symptoms in anxious MDD (Jaeckle et al., 2021). Consistent with this, Jaeckle et al. (2021) found that the anxious MDD group exhibited higher levels of anger towards others relative to the non-anxious MDD group, thus displaying a lower selectivity for biases toward self- versus other-blame-related feelings. In contrast, non-anxious MDD patients benefited from neurofeedback relative to the solely psychological intervention and were less likely to report other-directed anger (Jaeckle, 2018, Jaeckle et al., 2021). This led us to conceptualise self-blame-selective RSATL-BA25 hyper-connectivity as a marker of non-anxious MDD with favourable short-term prognosis for symptom improvement, but higher risk of recurrence.

## 2 Materials and Methods

### 2.1 Participants

Participants were partly recruited from the cluster-randomised Antidepressant Advisor trial (ADeSS; NCT03628027), which evaluated the feasibility of a novel computerised decision support algorithm for antidepressant medications in MDD patients in primary care (Harrison et al., 2020, Harrison et al., 2022). Participants enrolled in the ADeSS trial were assigned to either i) use of a computerised decision-support tool by their general practitioner (GP) to assist with antidepressant choices, or ii) treatment-as-usual, and were asked to attend an optional MRI session. Most participants, however, were recruited outside of the ADeSS trial through online advertising and resembled the treatment-as-usual arm. Ethical approval was obtained from the NHS Health Research Authority and National Research Ethics Service London – Camberwell St Giles Committee (REC reference: 17/LO/2074). All participants provided informed, written consent and received compensation for their time and for their travel expenses.

Participants in the MDD group fulfilled criteria for a MDD according to the Structured Clinical Interview for DSM-5 (First et al., 2015), were currently experiencing a major depressive episode (MDE), and had at least moderately severe depressive syndrome on the Patient Health Questionnaire (Spitzer et al., 1999) (PHQ-9; score ≥ 15). Moreover, they were non-responders to at least two serotonergic antidepressants from the following list in current or previous episodes: citalopram, fluoxetine, sertraline, escitalopram, paroxetine, venlafaxine, or duloxetine. All participants were encouraged to optimise their medication and followed-up after four months in primary care. Prior to their medication review, participants completed an fMRI paradigm, in which they viewed self- and other-blaming emotion-evoking statements.

Age- and gender-matched control participants without a history of MDD, but including anxiety disorders, and scoring below 10 on the PHQ-9 depression scale were recruited outside of the ADeSS trial through online advertising and asking enrolled participants with MDD to refer their friends and family. After the initial assessment, control participants completed the same fMRI paradigm which allowed for further interpretation and exploratory cross-sectional comparisons with the MDD group (not pre-registered). For more information about inclusion/exclusion criteria, recruitment, clinical assessment, and measures collected, please see Supplementary Methods.

We considered three analytical samples. For the primary imaging analysis, we included 34 participants with current MDD. All met strict criteria for signal dropout (sufficient coverage of the bilateral subgenual cingulate cortex) and movement (per-acquisition translation < 6 mm; per-acquisition rotation < 2 degrees; <25% censored volumes). For the secondary imaging analysis, we additionally included five participants who did not meet the strictest fMRI quality control threshold (“reserve list”) to assess the impact of lower fMRI quality on the findings, giving a total of 39 participants. Finally, for exploratory cross-sectional analyses to help with interpretation, we compared the MDD group with 15 control participants (13 of whom met the strict criteria and two additional control participants who did not meet the strictest criteria, “reserve list”; Supplementary Table 1).

### 2.2 Primary outcome

As stated in our pre-registered protocol (NCT04342299), we used a continuous measure of clinical outcome rather than categorising participants into responders and non-responders using the standard definition of a 50% reduction in scores (Nierenberg and DeCecco, 2001) as assessed by the self-rated Quick Inventory of Depressive Symptomatology (16-item; QIDS-SR16) (Rush et al., 2003) after four months of primary care treatment-as-usual, due to an unbalanced split between the resulting groups. Clinical outcome was determined by computing the percentage change from baseline to follow-up on our pre-registered primary outcome measure, the self-reported QIDS-SR16, where negative percentages corresponded to a reduction in depressive symptoms.

### 2.3 fMRI acquisition and paradigm

**Supplementary figure 1:**
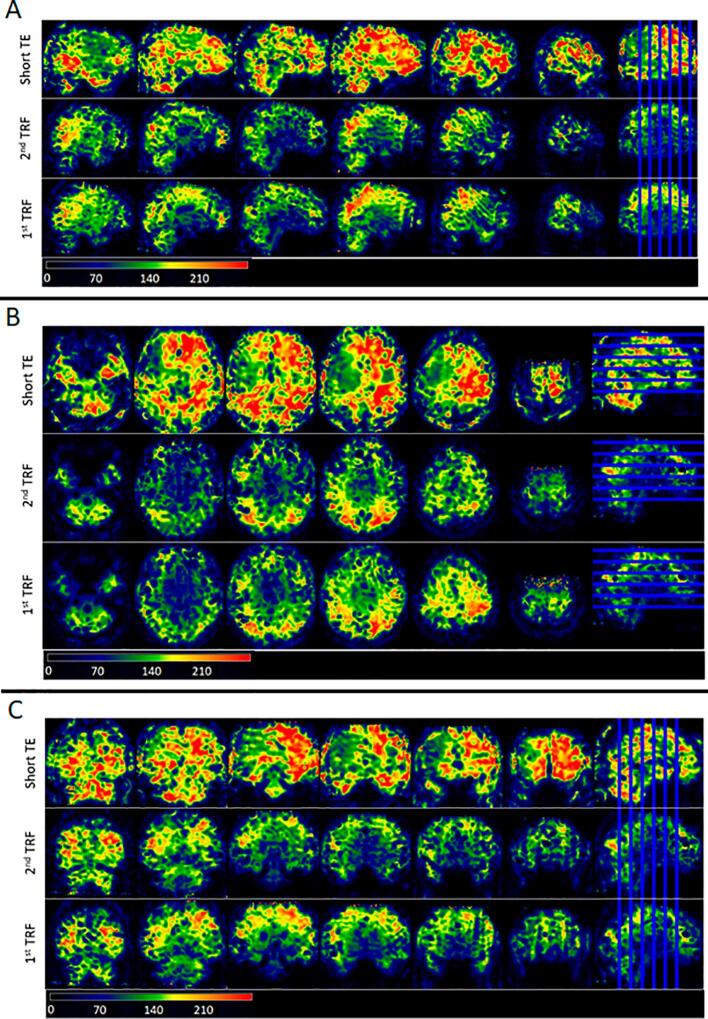
**Pilot data of one volunteer used to determine temporal signal-to-noise ratios (tSNRs).** There were three separate sessions of the optimised value-related moral sentiment task, either using a tailored radio frequency (TRF) sequence (twice) or a short time-echo (TE) sequence. Overall, the short TE sequence had a better tSNR compared to the TRF sequence (session 1 and 2). However, both sequences exceeded the minimum threshold of 40 for most regions as proposed by Murphy et al. (2007). Panel A shows a sagittal view, Panel B shows an axial view, and Panel C shows a coronal view. The colour bar represents tSNR values. Displayed using MRIcron (Rorden & Brett, 2000).

We used an fMRI protocol (MR750 3 T MR system; GE Healthcare) optimised for detection of ventral brain regions (Supplementary Methods). As demonstrated by measurements of the temporal signal-to-noise, i.e. “the mean of a voxel’s blood-oxygen level-dependent (BOLD) signal over time divided by its standard deviation over time” (Welvaert et al., 2013), overall signal quality was very good (Supplementary Table 2; Supplementary Fig. 1).

Participants were shown an optimised and shortened version of the fMRI paradigm outlined by Green et al. (2012) and Lythe et al. (2015), where we have established that the comparison between self- and other-agency conditions captures self-blaming emotional biases in post-scanning ratings, whilst controlling for overall negative valence and response time, as well as self- and other-reference (Zahn et al., 2009). For details on the optimisation, please refer to Duan et al., 2023 and Fennema, 2022. In brief, participants were shown 54 short written statements describing actions counter to social and moral values described by social concepts (e.g. impatient, dishonest) in which the agent was either the participant (self-agency condition [n = 27]) or their best friend (other-agency condition [n = 27]). In addition, there were 27 low-level null events as baseline condition (i.e. fixation of a visual pattern with no button press or other response required).

Participants also completed a previously validated computerised task (“Moral sentiment and action tendencies” (MSAT) (Duan et al., 2022, Duan et al., 2023, Fennema, 2022, Jaeckle et al., 2021)), which presented the same stimuli as the fMRI paradigm, to elucidate the neurocognitive underpinning of blame-related emotions. They were asked to select the emotion that best described how they would feel given the unpleasant hypothetical situation (guilt, shame, contempt/disgust towards self, contempt/disgust towards friend, indignation/anger towards friend, or no feeling/other feeling) and indicate how strongly they would blame themselves and their friend for the imagined behaviour. See Supplementary Methods for more details.

### 2.4 Image analysis

Following standard Statistical Parametric Mapping (SPM12; https://www.fil.ion.ucl.ac.uk/spm12) pre-processing steps, additional motion correction was applied in the form of censoring, i.e. identifying outliers based on framewise displacement and regressing them from the fMRI timeseries (Power et al., 2012, Power et al., 2014) (see Supplementary Methods), to compensate for using fairly lenient translation and rotation cut-offs given our patient population. Voxel-based analyses were Family-Wise Error (FWE) corrected at *p* =.05 at the voxel-level over the whole brain and using small-volume correction over our two pre-registered *a priori* regions-of-interest (ROIs; further described below).

To test our pre-registered hypotheses, connectivity was determined using psychophysiological interaction (PPI) analysis. We extracted the signal from our pre-registered seed region, i.e. the right superior anterior temporal lobe (RSATL; MNI coordinates: x  = 58, y = 0, z = -12; 6 mm sphere), and created interaction terms for the psychological variable (main effect of condition, i.e. self-agency vs fixation and other-agency vs fixation) with the physiological variable (the RSATL signal time course irrespective of condition).

We conducted a one-sample *t*-test at the second level on the PPI contrast maps for self- vs other-blaming emotions to test whether the regression coefficient for QIDS-SR16 change modelled as a covariate was different from zero. Two pre-registered ROIs were used for small volume correction, i.e. posterior subgenual cortex (BA25; cluster derived from Lythe et al., 2015) and right striatum / pallidum (right hemispheric part of the basal ganglia, described in Lawrence et al., 2022). An exploratory analysis, using the same *a priori* ROIs, examined whether BOLD activation was associated with clinical outcomes. Regression coefficient averages over the pre-registered *a priori* ROIs were also extracted for individual participants, for each contrast (self-agency vs fixation, other-agency vs fixation and the subtraction-based difference), using the MarsBaR toolbox (Brett et al., 2002). These were further explored in IBM SPSS Statistics 27.

Lastly, exploratory second-level BOLD and PPI analyses were conducted to examine differences in self-blaming emotional biases between participants with MDD and controls using small volume correction over our pre-registered *a priori* ROIs to support the interpretation of prognostic effects. For more details, please see Supplementary Methods. All analyses were inclusively masked with a grey matter mask as previously described in Green et al. (2012).

### 2.5 Behavioural data analysis

All data analyses were carried out using IBM SPSS Statistics 27, using a significance threshold of *p* =.05, two-tailed. Data were checked for outliers, using standardised scores (outside *z* = ± 2.5 standard deviations from the mean). Correlation analysis (Spearman’s rho) was used to investigate the association between the pre-registered neural signatures and QIDS-SR16 change, as well potential clinical confounders. For details on the processing and analysis of the computerised MSAT task, please see Supplementary Methods.

## 3 Results

### 3.1 Subgroup characteristics

MDD and control groups did not differ significantly on demographic variables (Supplementary Table 3). There was also no evidence for a difference in terms of movement during fMRI or response times (Supplementary Table 4). However, the MDD group perceived the other-blame condition as “quite unpleasant” more often compared with controls (57% vs 41%, respectively; *t*(51) = 3.01, *p* =.004). The computerised MSAT task showed that most statements were able to evoke moderate to strong feelings of self- and other-blame, while also revealing significant differences in blame attribution and self-contempt bias between the MDD group and controls (Supplementary Table 5; Supplementary Findings).

Baseline clinical characteristics of participants with MDD are shown in Table 1 (for control participants, see Supplementary Table 6). Most participants with MDD fulfilled the DSM-5 anxious distress specifier criteria (77%), often combined with atypical features (46%). Moreover, many MDD participants met criteria for a life-time axis I co-morbidity (87%), with posttraumatic stress disorder (PTSD; 44%) and other anxiety disorder (41%) as most common co-morbidities.

**Table 1:** Baseline clinical characteristics MDD (n = 39).

**MDD modified DSM-5 subtype**	**No. (%)**

Anxious distress only	7 (18%)
Melancholic features only	0 (0%)
Melancholic features + anxious distress	5 (13%)
Atypical features only	2 (5%)
Atypical features + anxious distress	18 (46%)
No specific subtype	7 (18%)
**Age of depression onset (in years), M ± SD; min–max**	17.9 ± 8.9; 4 – 42
**Current MDE duration (in months), M ± SD; min–max**	26.6 ± 45.4; 1 – 176
**Number of MDEs, M ± SD; min–max**	6.6 ± 4.9; 1 – 20
**Illness duration (in years), M ± SD; min–max**	25.0 ± 15.8; 2 – 56
**Number of suicide attempts, M ± SD; min–max**	0.6 ± 1.4; 0 – 6
**Maudsley Staging Method**	
Mild	16 (41%)
Moderate	23 (59%)
Severe	0 (0%)
**Life-time axis-I co-morbidity**	
Posttraumatic stress disorder	17 (44%)
Other anxiety disorder	16 (41%)
Obsessive-compulsive disorder	3 (8%)
Eating disorder	14 (36%)
None	5 (13%)
**Family history**	
First degree relative with MDD	14 (36%)
First degree relative with bipolar disorder	2 (5%)
No family history of MDD	18 (46%)

Percentages may not add up to 100 due to rounding. MDD = major depressive disorder; DSM-5 = Diagnostic and Statistical Manual for Mental Disorders 5th edition; MDE = major depressive episode; M: mean; SD: standard deviation; min = minimum; max = maximum.

As part of the study, participants were encouraged to optimise their antidepressant medication, which often was a selective serotonin reuptake inhibitor (SSRI; 82%; Supplementary Table 7). However, more than half (54%) did not change their medication and some even stopped their medication (16%; Supplementary Table 8). On average, participants showed a reduction in depressive symptoms from baseline to follow-up, both self- and observer-rated (Table 2). The percentage change in QIDS-SR16 was consistent regardless of medication status (i.e. no change in medication, minimal change or relevant change; *F*(2,36) = 1.44, *p* =.25), or any of the other clinical measures at baseline (Supplementary Table 9). However, there was a positive association between current MDE duration and percentage change in QIDS-SR16 (*r*_s_(34) = 0.42, *p* =.01), showing that those with a longer current MDE duration had poorer clinical outcomes.

**Table 2:** Descriptive statistics for clinical symptom measures at baseline and follow-up MDD (n = 39).

	Baseline (mean ± SD; min – max)	Follow-up (mean ± SD; min – max)	Difference [95% CI]
QIDS-SR16	17.4 ± 3.5; 10 – 23	13.1 ± 5.6; 4 – 24	−4.3 [-6.1, −2.6]
MM-PHQ-9	18.7 ± 4.6; 8 – 27	13.7 ± 7.9; 0 – 27	−5.0 [-7.3, −2.8]
GAD-7 ^a^	11.3 ± 4.3; 1 – 21	10.1 ± 5.8; 0 – 21	−1.3 [-3.2, 0.6]
MADRS	31.5 ± 4.9; 22 – 42	23.3 ± 11.2; 3 – 44	−8.2 [-11.2, −5.1]
SOFAS	53.6 ± 5.4; 33 – 61	58.6 ± 11.2; 33 – 85	5.0 [2.1, 7.9]

^a^ Missing follow-up data for one participant.

MDD = major depressive disorder; CI = confidence interval; QIDS-SR16 = Quick Inventory of Depressive Symptomatology – self-rated, 16 items; MM-PHQ-9 = Maudsley Modified Personal Health Questionnaire, 9 items; GAD-7 = Generalised Anxiety Disorder, 7 items; MADRS = Montgomery-Åsberg Depression Rating Scale; SOFAS = Social and Occupational Functioning Assessment Scale. M = mean; SD = standard deviation; min = minimum; max = maximum.

### 3.2 fMRI findings

**Fig. 1:**
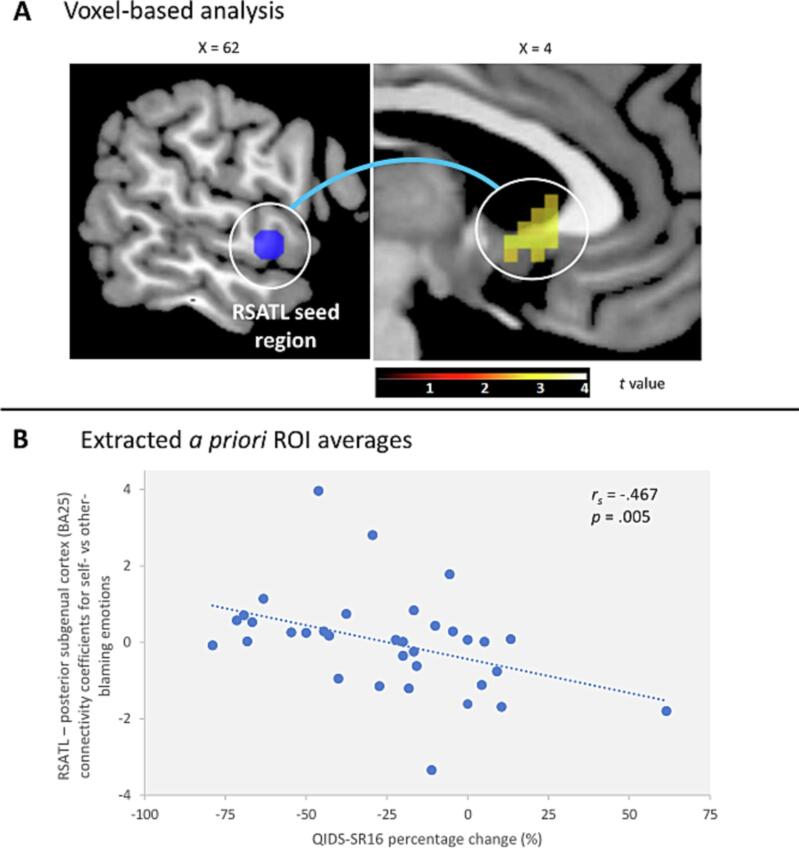
**Connectivity between right superior anterior temporal lobe (RSATL) seed region and posterior subgenual cortex (BA25) for self-blaming vs other-blaming emotions.** Panel (A) shows the enhanced PPI effect from the voxel-based analysis for RSATL-posterior subgenual cortex (BA25) connectivity as a cropped section through the posterior subgenual cortex (BA25), displayed using MRIcron (Rorden and Brett, 2000) at an uncorrected voxel-level threshold of *p* =.005, with no cluster-size threshold (the colour bar represents *t* values; the numbers above the brain slices stand for x-coordinates of the Montreal Neurological Institute coordinate system). Panel (B) shows a negative association between RSATL-posterior subgenual cortex (BA25) connectivity and QIDS-SR16 percentage change from baseline to follow-up, using the extracted *a priori* posterior subgenual cortex (BA25) ROI averages. Higher connectivity for self-blaming vs other-blaming emotions is associated with more negative QIDS-SR16 scores, i.e. improvement of depressive symptoms. RSATL = right superior anterior temporal lobe; BA = Brodmann Area; QIDS-SR16 = Quick Inventory of Depressive Symptomatology - self-rated, 16-items; *r_s_* = Spearman correlation; PPI = psychophysiological interaction; ROI = region-of-interest.

**Supplementary figure 2:**
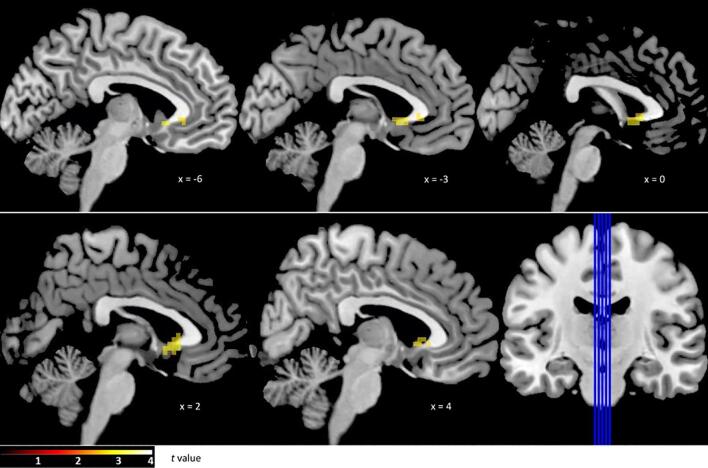
**Sagittal view of connectivity between right superior anterior temporal lobe seed region and posterior subgenual cortex (BA25) for self-blaming vs other-blaming emotions.** The panel shows sagittal slices of the significant posterior subgenual cortex (BA25) cluster, displayed using MRIcron (Rorden & Brett, 2000) at an uncorrected voxel-level threshold of *p* = .005, with no cluster-size threshold (the colour bar represents *t* values; the numbers below the brain slices stand for x-coordinates of the Montreal Neurological Institute coordinate system). BA = Brodmann Area.

**Supplementary figure 3:**
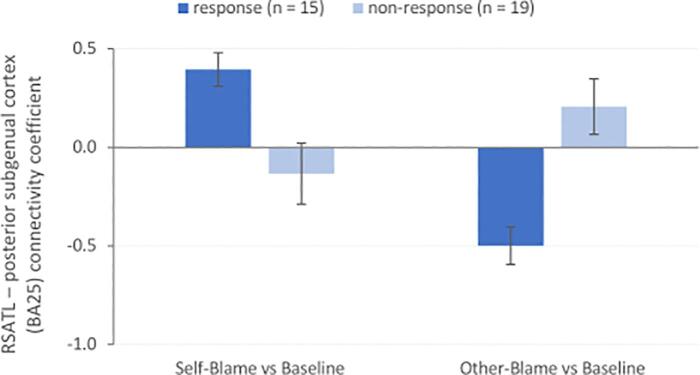
**Interaction effect between group (partial responders vs non-responders) and condition (self- vs other-blaming) for RSATL – posterior subgenual cortex (BA25) connectivity.** There was an interaction effect between group (partial responders vs non-responders, where partial responder was defined as participants who showed at least a 25% reduction in depressive symptoms as measured on the QIDS-SR16) and condition (self- vs other-blaming) for RSATL – posterior subgenual cortex (BA25) connectivity, using the extracted *a priori* posterior subgenual cortex (BA25) ROI averages. The interaction effect was driven by higher RSATL – posterior subgenual cortex (BA25) connectivity during self-blame in the partial response group compared to the non-response group, and lower RSATL – posterior subgenual cortex (BA25) connectivity during other-blame in the partial response group compared to the non-response group. There was a difference between groups on self-blame vs other-blame differences, which was identified by the observed interaction effect. RSATL = right superior anterior temporal lobe; BA = Brodmann Area; QIDS-SR16 = Quick Inventory of Depressive Symptomatology – self-rated, 16 items; ROI = region-of-interest.

As hypothesised (Hypothesis 1), the posterior subgenual cortex (BA25) and RSATL exhibited higher connectivity for self- vs other-blame in patients with larger reductions in symptoms after four months, i.e. favourable clinical outcomes (Table 3). This RSATL-posterior subgenual cortex (BA25) hyper-connectivity was confirmed when extracting the *a priori* posterior subgenual cortex (BA25) average PPI effect for self- vs other-blame (*r_s_*(34) = -0.47, *p* =.005; Fig. 1; Supplementary Figure 2; Supplementary Figure 3; Supplementary Findings) and remained when excluding potential outliers (*r_s_*(32) = -0.45, *p* =.01) as well as when including the reserve list (*r_s_*(39) = -0.32, *p* =.05). There was no association between the only potential confounder, current MDE duration, and the neural signature (*r_s_*(34) = -0.25, *p* =.16). In contrast, there was no evidence for a PPI effect or association with QIDS-SR16 change for our other pre-registered ROI (Hypothesis 2), i.e. right striatum / pallidum (*r_s_*(34) = -0.16, *p* =.37). The whole-brain PPI analysis did not reveal any additional regions, nor did our exploratory voxel-based BOLD effect analysis reveal any activations associated with clinical outcomes.

**Table 3:** RSATL psychophysiological interaction effects for self- vs other-blaming emotions (n = 34).

				*MNI peak coordinates*				
Hemi-sphere	Region	Cluster size	Brodmann Area	x	y	z	*t* statistic	Voxel-based FWE-corrected *p* value
Negative association QIDS-SR16 change:								
left	Posterior subgenual cingulate	37	25	−3	17	−4	3.03	.021^a^
Positive association QIDS-SR16 change:								
NA	No significant regions	NA	NA	NA	NA	NA	NA	NA

^a^ Region surviving voxel-based FWE correction over *a priori* posterior subgenual cortex (BA25) region of interest (6 mm sphere, MNI: x  = 2, y = 14, z = -6, (Lythe et al., 2015).

RSATL = right superior anterior temporal lobe; QIDS-SR16 = Quick Inventory of Depressive Symptomatology – self-rated, 16 items; MDD = major depressive disorder; FWE = Family-Wise Error; MNI = Montreal Neurological Institute.

**Supplementary figure 4:**
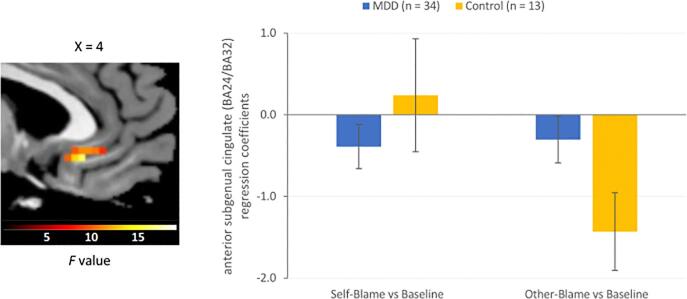
**BOLD interaction effect between group (MDD vs control) and condition (self- vs other-blaming) in the right anterior subgenual cingulate cortex (BA24/BA32).** A cropped section through the right anterior subgenual cingulate cortex (BA24/BA32), displayed using MRIcron (Rorden & Brett, 2000) at an uncorrected voxel-level threshold of *p* = .005, with no cluster-size threshold (the colour bar represents *F* values; the number above the brain slice stand for the x-coordinate of the Montreal Neurological Institute coordinate system). This interaction was due to lower anterior subgenual cingulate (BA24/BA32) signal for other-blame in the control group compared to the MDD group, and higher anterior subgenual cingulate (BA24/BA32) signal for self-blame in the control group compared to the MDD group. There was a main effect of condition, but no main effect of group. Anterior subgenual cingulate (BA24/BA32) regression coefficients were derived from the whole-brain analysis cluster. MDD = major depressive disorder; BOLD = blood-oxygen level-dependent; BA = Brodmann Area.

The exploratory cross-sectional analyses revealed a BOLD interaction effect between group (MDD vs control) and condition (self- vs other-blaming emotions) for the right anterior subgenual cingulate (BA24/BA32; *F* = 9.96, voxel-based FWE-corrected *p* =.047; no multiple comparison correction for the number of *a priori* ROIs = 4 was applied, which would have rendered this finding statistically insignificant; Supplementary Results; Supplementary Table 10). Contrary to our previous study in medication-free remitted MDD (Lythe et al., 2020), this was driven by lower anterior subgenual cingulate (BA24/BA32) signal for other-blame in the control group compared with the MDD group, and higher anterior subgenual cingulate (BA24/BA32) signal for self-blame in the control group compared with the MDD group (Supplementary Figure 4). Interestingly, MDD patients with higher anxiety symptoms showed lower anterior subgenual cingulate (BA24/BA32) signal during the self-blame condition (*r_s_*(34) = −0.35, *p* =.05).

## 4 Conclusions

### 4.1 Discussion

We corroborated our first pre-registered hypothesis (Hypothesis 1), that self-blame-selective hyper-connectivity between the RSATL and the posterior subgenual cortex (BA25) is prospectively associated with favourable clinical outcomes in current MDD. No other brain regions were revealed in our whole brain analysis. To our knowledge, this is the first demonstration of self-blame-related neural signatures as prognostic predictors in current MDD and shows the relevance of this signature previously identified in remitted MDD at high risk of recurrence (Lythe et al., 2015). One potential explanation is that this neural signature serves as a treatment target and is amenable to modification by treatment, thereby improving clinical outcomes, similarly, to normalised subgenual frontal metabolism (Drevets et al., 2002, Drevets et al., 1997, Holthoff et al., 2004, Mayberg et al., 2000). Alternatively, in analogy to previous proposals for subgenual frontal activations, self-blame-selective hyper-connectivity may be required to facilitate response to treatment rather than serve as the target itself; this adaptive mechanism could be lacking in non-responders (Mayberg et al., 1997, Roiser et al., 2012). Given that the majority of our MDD group did not change treatment, however, we think that it is more plausible that self-blame-selective RSATL-BA25 hyper-connectivity may reflect a subtype of depression with a higher likelihood of spontaneous improvement.

Contrary to our second pre-registered hypothesis (Hypothesis 2), we found no association between RSATL-ventral striatum connectivity and clinical outcomes in current MDD. This indicates that this neural signature is less likely to be relevant for clinical outcomes in treatment-resistant current MDD (Ming et al., 2017). We were unable to investigate our third pre-registered hypothesis (Hypothesis 3) that patients with anxious distress showed a lack of self-blame-selective RSATL-posterior subgenual cortex (BA25) hyper-connectivity, and thus poorer clinical outcomes, because our sample predominantly consisted of anxious MDD. However, our exploratory cross-sectional analyses showed that anxiety symptoms did indeed reduce self-blame-selective anterior subgenual cingulate activation at baseline, thus reversing a pattern of activation we had previously found in this area in fully remitting MDD with low levels of co-morbidity (Lythe et al., 2015). This is in keeping with the hypothesis that the neural architecture of blame-related feelings differs between non-anxious and anxious forms of MDD, the latter being predominant in populations with treatment-resistant depression (Dold et al., 2017, Domschke et al., 2010, Gaspersz et al., 2017). This has important implications for the stratification of neuromodulation treatments of these neural systems when tackling self-blaming feelings in these patients (Jaeckle et al., 2021), which were also demonstrated at the behavioural level by showing increased self-contempt biases and agency-incongruent self-blaming biases compared to the control group. The latter refers to blaming oneself for other people’s wrongdoings and is characteristic of the overgeneralised and over-responsible nature of depression-typical guilt (O'Connor et al., 2002).

### 4.2 Limitations

MDD is an inherently heterogeneous disorder, resulting in patients with a wide variety of symptoms, natural courses, and treatment responses (Strawbridge et al., 2017). The current study represents a particularly heterogeneous sample by allowing for co-morbid non-psychotic axis-I disorders, apart from alcohol and substance use disorders. This makes it hard to disentangle the contribution of co-morbidities to self-blaming biases. For example, PTSD has also been associated with disrupted emotional processing in the form of hyper-activation of the amygdala and hypo-activation of the ventromedial prefrontal cortex (Hayes et al., 2012, Pitman et al., 2012).

However, we aimed to evaluate the prognostic value of blame-related neural signatures in a pragmatic clinical setting, which reflects the high co-morbidity between MDD and anxiety disorders (Godlewska, 2020). We previously employed stricter inclusion and exclusion criteria to aid with the identification of distinctive features of MDD. However, this approach limited the generalisability to clinical populations and thus potential clinical utility (Kapur et al., 2012). Moreover, co-morbidities themselves can contribute to clinical outcomes, which is particularly evident with the reported association between co-morbid anxiety disorders in MDD and poorer clinical outcomes (Dold et al., 2017, Domschke et al., 2010, Fava et al., 2008). Even though the current sample was recruited from primary care, it should be noted that it mostly consisted of chronic MDD patients, often with anxious distress and other co-morbidities which itself may represent a distinct patient group.

Another limitation concerns the relatively modest sample size of the current study, which, depending on fMRI quality control threshold used, fluctuated around the minimum recommend size of n = 35 for a pilot study to estimate effect size (Teare et al., 2014). Small studies can lead to biased estimates, low replicability, and a lack of adequate statistical power to detect small effect sizes (Teare et al., 2014, Turner et al., 2018). Future studies need to investigate whether the findings can be replicated in a larger sample of patients.

Lastly, treatment in our observational study was not standardised and included a mix of antidepressant medications (SSRIs, selective norepinephrine reuptake inhibitors (SNRIs) and tricyclics) and psychotherapy. It has been shown that different types of antidepressants, e.g. SSRIs, SNRIs, and norepinephrinergic and specific serotonergic antidepressants, have different effects on brain function (Bruhl et al., 2011, Frodl et al., 2011). Thus, medication effects may have introduced variability in the observed neural responses. However, it reflects primary care treatment-as-usual which can be considered as a complex multifaceted intervention and medication effects may contain prognostic information as well, e.g. whether there has been an effect at all.

### 4.3 Conclusion

This study shows the pathophysiological relevance of overgeneralised feelings of self-blame and their neural correlates in current and treatment-resistant MDD. We demonstrated that self-blame-selective hyper-connectivity between the RSATL and the posterior subgenual cortex (BA25) is relevant for clinical outcomes. Future studies need to investigate whether this neural signature represents a trait-like feature of a fully remitting subtype of MDD, or whether it is also modulated by depressive state and how it changes in response to pharmacological and psychological interventions.

## CRediT authorship contribution statement

**Diede Fennema:** Conceptualization, Methodology, Formal analysis, Investigation, Data curation, Writing – original draft, Visualization, Funding acquisition. **Gareth J. Barker:** Conceptualization, Methodology, Writing – review & editing, Supervision. **Owen O'Daly:** Conceptualization, Methodology, Writing – review & editing. **Suqian Duan:** Investigation, Writing – review & editing. **Ewan Carr:** Conceptualization, Methodology, Writing – review & editing. **Kimberley Goldsmith:** Conceptualization, Methodology, Writing – review & editing. **Allan H. Young:** Conceptualization, Methodology, Writing – review & editing, Supervision, Project administration, Funding acquisition. **Jorge Moll:** Conceptualization, Writing – review & editing. **Roland Zahn:** Conceptualization, Methodology, Formal analysis, Writing – review & editing, Supervision, Project administration, Funding acquisition.

## Declaration of Competing Interest

The authors declare the following financial interests/personal relationships which may be considered as potential competing interests: RZ is a private psychiatrist service provider at The London Depression Institute and co-investigator on a Livanova-funded observational study of Vagus Nerve Stimulation for Depression. RZ has received honoraria for talks at medical symposia sponsored by Lundbeck as well as Janssen. RZ has collaborated with EMOTRA, EMIS PLC and Depsee Ltd. RZ is affiliated with the D’Or Institute of Research and Education, Rio de Janeiro and advises the Scients Institute, USA. GB receives honoraria for teaching from GE Healthcare. AHY is employed by King’s College London as an honorary consultant in the South London and Maudsley Trust (NHS UK) and is a consultant to Johnson & Johnson and Livanova. AHY has given paid lectures and sat on advisory open access boards for the following companies with drugs used in affective and related disorders: Astrazenaca, Eli Lilly, Lundbeck, Sunovion, Servier, Livanova, Janssen, Allegan, Bionomics, Sumitomo Dainippon Pharma. Prof Young has received honoraria for attending advisory boards and presenting talks at meetings organised by LivaNova. AHY is the Principal Investigator of the following studies: Restore-Life VNS registry study funded by LivaNova, ESKETINTRD3004: ‘An Open-label, Long-term, Safety and Efficacy Study of Intranasal Esketamine in Treatment-resistant Depression’, ‘The Effects of Psilocybin on Cognitive Function in Healthy Participants’ and ‘The Safety and Efficacy of Psilocybin in Participants with Treatment-Resistant Depression (P-TRD)’. AHY has received grant funding (past and present) from the following: NIMH (USA); CIHR (Canada); NARSAD (USA); Stanley Medical Research Institute (USA); MRC (UK); Wellcome Trust (UK); Royal College of Physicians (Edin); BMA (UK); UBC-VGH Foundation (Canada); WEDC (Canada); CCS Depression Research Fund (Canada); MSFHR (Canada); NIHR (UK); Janssen (UK). AHY has no shareholdings in pharmaceutical companies. KG reports grants from NIHR, Stroke association, National Institutes of Health (US) and Juvenile Diabetes Research Foundation (US) during the conduct of the study. EC reports personal fees from NIHR during the conduct of the study. None of the other authors report biomedical financial interests or potential conflicts of interested related to the subject of this paper.

## Data Availability

Data will be made available on request.
