# Supplementary material for: Self-blame-selective hyper-connectivity between anterior temporal and subgenual cortices predicts prognosis in major depressive disorder
Source: Neuroimage Clin. 2023 Jun 16;39:103453. doi: 10.1016/j.nicl.2023.103453 (PMC10336192; doi:10.1016/j.nicl.2023.103453)
Supplement: Supplementary data 1 [file mmc1.docx]

**SUPPLEMENTAL INFORMATION**

**Self-blame-selective hyper-connectivity between anterior temporal and subgenual cortices predicts prognosis in major depressive disorder**

Diede Fennema^1^, Gareth J. Barker^2^, Owen O’Daly^2^, Suqian Duan^1^, Ewan Carr^3^, Kimberley Goldsmith^3^, Allan H. Young^1,5^, Jorge Moll^4^ & Roland Zahn^1,4,5*^

*^1^ Centre of Affective Disorders, Institute of Psychiatry, Psychology & Neuroscience, Centre for Affective Disorders, King’s College London, London, UK*

*^2^ Department of Neuroimaging, Institute of Psychiatry, Psychology & Neuroscience, King’s College London, London, UK*

*^3^ Department of Biostatics and Health Informatics, Institute of Psychiatry, Psychology & Neuroscience, King’s College London, London, UK*

*^4^ Cognitive and Behavioural Neuroscience Unit, D’Or Institute for Research and Education (IDOR), Rio de Janeiro, Brazil*

*^5^ National Service for Affective Disorders, South London and Maudsley NHS Foundation Trust, London, UK*

* Corresponding author

Professor Roland Zahn (see address above)

E-mail: roland.zahn@kcl.ac.uk

Phone: 0044-(0)20 7848 0348

Fax: 0044-(0)20 7848 0298

Keywords: *fMRI; self-blame; depression; biomarker; prognosis*

# Supplementary Methods

## *Additional exclusion criteria*

General exclusion criteria were: previous prescription of mirtazapine or vortioxetine at therapeutic dose, MRI contraindications, currently receiving specialist psychiatric treatment, high suicide risk on the Mini International Neuropsychiatric Interview (MINI) suicidality screen (Sheehan et al., 1998), past diagnosis of schizophrenia or schizo-affective disorder, psychotic symptoms using clinical screening questions, bipolar disorder, at risk of being violent, drug or alcohol abuse over the last six months, suspected neurological condition, pregnancy or insufficient contraception in women of childbearing age and breastfeeding or within six months of giving birth.

## *Recruitment and clinical assessment*

We recruited participants from September 2018 to March 2020 partly through from a cluster-randomised feasibility clinical trial, the Antidepressant Advisor Study (ADeSS; NCT03628027). Recruitment was halted due to the COVID-19 pandemic and recommenced in October 2020 using online advertising only and was completed in August 2021.

As described in the trial protocol (Harrison et al., 2020), general practitioner (GP) practices screened for patients with a history of treatment-resistance to antidepressant medications within their practice, i.e. non-responders to at least two serotonergic antidepressants in the current or previous episodes. Potential participants were approached for consent and, if given, asked to fill in a pre-screening questionnaire. Potentially eligible participants were invited for an in-depth assessment by the study team, which included a clinical assessment using the Structured Clinical Interview for DSM-5 (SCID) to establish a current major depressive disorder (MDD) (First, Williams, Karg, & Spitzer, 2015), a history of participants’ depressive episodes, their current and past antidepressant medications, and completing various clinical, behavioural and experimental measures.

A follow-up assessment was conducted to establish whether any changes in baseline measures had occurred. This visit took place around 14-18 weeks after enrolling in the study, which should allow observation of any treatment effect if there is one. It covered questions related to medications taken in the study period as well as various clinical and behavioural measures. The main clinical measures collected at baseline and follow-up were the Quick Inventory of Depressive Symptomology (16 items, self-rated; QIDS-SR16) (Rush et al., 2003), Maudsley Modified Patient Health Questionnaire (9 items; MM-PHQ-9) (Harrison et al., 2021), Generalised Anxiety Disorder (7 items; GAD-7) (Spitzer, Kroenke, Williams, & Lowe, 2006), Montgomery-Åsberg Depression Rating Scale (MADRS) (Montgomery & Asberg, 1979), and Social and Occupational Functioning Assessment Scale (SOFAS, part of SCID) (First et al., 2015). Please refer to the ADeSS trial protocol for more details regarding these procedures (Fennema, 2022; Harrison et al., 2020).

As the ADeSS trial was stopped due to the COVID-19 pandemic, an alternative recruitment route was employed to continue recruitment for the fMRI study. Trial adverts were posted online, with further dissemination of study adverts via university and institutional recruitment circulars. Interested participants were asked to complete a similar pre-screening questionnaire as those approached for the ADeSS trial. If potentially eligible, participants were invited for an in-depth assessment to confirm their eligibility. For more details, please see Fennema (2022).

A total of 1,755 participants with a history of MDD showed interest in participating and completed a pre-screening questionnaire. Potentially eligible MDD participants (n = 89) for the ADeSS trial and the fMRI study were invited to attend an in-depth assessment. Of those, 45 participants enrolled in the fMRI study, attended their MRI session and completed the study. Of those 45 participants, ten participants were also part of the ADeSS trial (support tool arm: n = 4; treatment-as-usual arm: n = 6).

Upon study completion, participants in the MDD group were asked to refer partners or friends who might be interested in serving as control participants. Moreover, trial adverts were posted online, with further dissemination of study adverts via university and institutional recruitment circulars. Interested participants were asked to complete a pre-screening questionnaire targeted to control participants. If potentially eligible, participants were invited for an in-depth assessment to confirm their eligibility and they completed a similar battery of clinical, behavioural and experimental measures as the MDD group.

A total of 350 control participants completed a pre-screening questionnaire, with n = 113 meeting the initial eligibility criteria. Twenty-four control participants were invited for the initial baseline. Following the assessment, n = 22 control participants were enrolled in the study (n = 3 referred by a participant in the MDD group) and n = 20 control participants attended their MRI session.

## *Additional experimental neuropsychological task*

In addition to the standard tests, participants completed an experimental, computer-based cognitive task, which investigates the neurocognitive underpinnings of blame-related emotions: the moral sentiment and action tendencies task (MSAT). This task has been validated in previous studies (Duan, Lawrence, Valmaggia, Moll, & Zahn, 2022; Green, Moll, Deakin, Hulleman, & Zahn, 2013; Jaeckle, 2018) but here, we used the modified, shortened version as described in Duan, Valmaggia, Fennema, Moll, and Zahn (2023).

Participants were shown the same stimuli as the fMRI moral sentiment paradigm using Excel Macro or using an online-based version on PsychoPy (Peirce et al., 2019). They were asked to select the emotion that best described how they would feel given the unpleasant hypothetical situation: guilt, shame, contempt/disgust towards self, contempt/disgust towards friend, indignation/anger towards friend, or no feeling/other feeling. Moreover, they were asked to select the action they would most strongly feel like doing: creating distance from self, hiding, apologising, creating distance from friend, verbally or physically attacking/punishing friend, or no action/other action. Lastly, participants had to indicate how strongly they would blame themselves (i.e. self-blame rating) and how strongly they would blame their friend (i.e. other-blame rating) for the imagined behaviour, using a 7-point visual analogue scale, where 1 = not at all and 7 = very much.

## *Sample size*

A formal power calculation was difficult, with no previous study from which effect sizes could be drawn. As such, this study should be considered as a proof-of-concept for using fMRI to prospectively predict prognosis in MDD. If the neural signatures have at least 70% accuracy, a minimum of n = 44 MDD patients is required to achieve 85% power for a significant prediction of response (*p* = .05) compared to chance (50%) using a binomial test. Even though a clinically relevant biomarker should show at least 80% accuracy (Savitz, Rauch, & Drevets, 2013), the proposed sample size is sufficient to determine the feasibility in a subsequent larger sample.

## *Optimisation imaging acquisition*

Temporal signal-to-noise ratio (tSNR) is not the same across all voxels in the brain – some regions will have higher or lower tSNR depending on their spatial location and corresponding T2* (Bennett & Miller, 2010). Signal drop-out is particularly profound in brain areas adjacent to bone and air cavities, such as orbitofrontal and inferior temporal regions (Ojemann et al., 1997). Even though these areas are typically implicated in emotional and/or moral social concepts relevant to the psychopathology of MDD, most studies fail to obtain sufficient coverage and thus dismiss clinically relevant areas (Zahn, de Oliveira-Souza, & Moll, 2020).

As such, we piloted two sequences to determine the best tSNR for the moral sentiment fMRI paradigm: a tailored radio frequency (TRF) sequence using hyperbolic secant pulses for signal excitation, based on work by Wastling and Barker (2015), and a short echo time (TE) sequence (20ms) as previously used for the fMRI paradigm (Green, Lambon Ralph, Moll, Deakin, & Zahn, 2012; Lythe et al., 2015). The TRF sequence was tested in two separate pilot sessions, while the short TE sequence was tested once. The tSNR for three sessions (short TE, TRF during the first pilot, and TRF during the second pilot) was calculated using the following formula [1]:

[1] $\frac{\bar{S}}{\sigma_{N}}$

where $\bar{S}$ is the mean activation signal of the fMRI time series and $\sigma_{N}$ the standard deviation of the noise in the time series. Raw values were extracted using the MarsBaR toolbox (Brett, Anton, Valabregue, & Poline, 2002) for the following regions-of-interest (ROI), identified as most relevant for self-blaming biases in MDD and generally hard to image:

1. Right superior anterior temporal lobe, as reported in Green et al. (2012). Montreal Neurological Institute (MNI) coordinates: x = 58, y = 0, z = -12; 6mm sphere.
2. Anterior subgenual cingulate cortex (BA24), as reported in Green et al. (2012). MNI coordinates: x = -4, y = 23, z = -5; 6mm sphere.
3. Posterior subgenual cortex (BA25), as reported in Lythe et al. (2015). MNI coordinates: x = 2, y = 14, z = -6; 6mm sphere.

## *MRI sequences*

Image acquisition was carried out on an MR750 3T MR system (GE Healthcare, Chicago, USA), using a Nova Medical 32-channel head coil. High-resolution anatomical images were acquired with an Inversion Recovery prepared Spoiled Gradient Echo sequence (repetition time (TR) = 7.3 sec; echo time (TE) = 3.0 sec; matrix = 256x256; flip angle = 11 degrees; field-of-view (FOV) = 270mm; slice thickness = 1.2mm, 196 slices). Images for incidental findings review were acquired using a Fast-Recovery Fast Spin-Echo (TR = 4380ms; TE = 64.85ms; matrix = 320x256; flip angle = 111 degrees; FOV = 240; slice thickness = 2mm, slice gap = 0mm, 72 slices) and Fluid-Attenuated Inversion Recovery sequence (TR = 8000ms; TE = 128.41ms; matrix = 256x128; flip angle = 111 degrees; FOV = 220; slice thickness = 4mm, slice gap = 0mm, 36 slices) and checked for brain abnormalities by a neuroradiologist at King’s College London Hospital, independent of additional, internal checks by the study team.

Functional image acquisition was obtained in the anterior commissure – posterior commissure plane, with slices running top to bottom, using a T2*-weighted echo-planar imaging blood-level oxygen-dependent (BOLD) sequence (repetition time = 2000ms; echo time = 20ms; matrix = 64x64; field-of-view = 211mm; flip angle = 75 degrees; slice thickness = 2.9mm, slice gap = 0.1mm, inter-slice distance = 3mm, 41 slices, 368 volumes). Shimming was automatically applied as part of the scanner’s “prescan” procedures, and four additional volumes were acquired and automatically discarded at the start of each fMRI run, allowing for T1 equilibration effects.

## *fMRI paradigm*

Stimuli were presented in an event-related design for a maximum of 5 seconds, within which time participants had to decide whether they would feel that the imagined behaviours were “quite unpleasant” or “mildly unpleasant” from their own perspective. The stimuli were presented in a pseudo-random order, presented at jittered intervals with a mean of 4000ms (with steps of 500ms). The total task time was 12 minutes and 9 seconds.

## *Image analysis*

Functional images were realigned, unwarped, and co-registered to the participant’s T1-weighted images. These images were normalised to MNI space using parameters derived from the unified segmentation of the T1-weighted image and resliced at a voxel size of 3x3x3mm. A smoothing kernel of full-width half-maximum equal to 6mm was used.

Framewise displacement was calculated using Brain and Mind Lab tools (https://github.com/spunt/bspm/blob/master/thirdparty/bramila/bramila_framewiseDisplacement.m). Any framewise displacement of $\geq$0.5mm was marked as a spike in movement and scan nulling regressors were added to the standard six motion parameter file to account for the spike(s). Participants with spikes in more than 25% of the functional images overall were deemed to have moved too much and were excluded from the analysis. Of the motion-contaminated volumes across the groups, 34% occurred in the self-agency condition, 35% in the other-agency condition, and 31% in the visual fixation condition.

At the individual level, BOLD effects were modelled for the self-agency condition, other-agency condition and null event, with an event duration of 0 seconds. Movement parameters (i.e. six parameters describing movement by rotation and translation in three dimensions each, plus any scan nulling regressors) were included as covariates. No time and dispersion derivatives were modelled as it was shown to result in lower internal reliability (Fennema, O'Daly, Barker, Moll, & Zahn, 2021). Contrasts were created to examine activation to self-blaming emotions (self-agency vs fixation), other-blaming emotions (other-agency vs fixation) and the subtraction-based difference between self- and other-blaming emotions.

Additional exploratory (not pre-registered) second-level BOLD and psychophysiological interaction (PPI) analyses were conducted to examine differences in self-blaming emotional biases between participants with MDD and controls, using a factorial model with two factors: group (MDD vs control) and condition (self- vs other-blame). Within the model set-up, no assumption of independence was made for condition, because both self- and other-blaming emotions were measured within the same participant. F-contrasts for main effects of group, condition and their interaction were thresholded at *p* = .001 (uncorrected voxel-level) and corrected for Family-Wise Error (FWE) at the voxel-level at *p* = .05 over *a priori* ROIs in two tiers. Tier 1 comprised our two pre-registered ROIs, namely the posterior subgenual cortex (BA25) derived from Lythe et al. (2015) (MNI: x = 2, y = 14, z = -6) and right striatum / pallidum, i.e. part of the right hemispheric basal ganglia (MNI: x = 21, y = 6, z = 4), as described in Lawrence et al. (2022).

Tier 2 ROIs consisted of the anterior subgenual cingulate (BA24; MNI coordinates: x = -4 / +4, y = 23, z = -5; 6mm sphere) and frontopolar cortex (BA10), both relevant to moral and emotional cognition (Zahn et al., 2020). The anterior subgenual cingulate (BA24) has been implicated in social agency and value of social outcomes (Moll et al., 2007; Moll, De Oliveira-Souza, & Zahn, 2008; Moll et al., 2006; Zahn et al., 2020), while frontopolar cortex (BA10) activation is consistently associated with guilt and is thought to represent long-term consequences of social behaviour (Wood & Grafman, 2003; Zahn et al., 2020; Zahn et al., 2017). In contrast to previous studies (Green et al., 2012; Lythe et al., 2020; Zahn et al., 2015), a bilateral anterior subgenual cingulate (BA24) ROI was used rather than to focus on the right hemispheric part, which was driven by the observed change in lateralisation of the peak of anterior subgenual cingulate (BA24) BOLD activation when using a different modelling approach (Fennema et al., 2021) as well as the notion of functional subdivisions in the subgenual region (Zahn et al., 2020).

## *Behavioural data analysis*

Data were checked for outliers using standardised scores (outside *z* = ± 2.5 standard deviations from the mean) for the MDD group and the control group separately. Results with outliers were confirmed by supplementary analyses replacing the outlying value by the nearest occurring value in the rest of the sample that was not an outlier. Moreover, data were screened for normal distribution within each group with Kolmogorov-Smirnov tests and if the assumption of normality was violated, non-parametric Mann-Whitney-*U* tests instead of independent sample t-tests were used to investigate between-group differences (MDD vs controls).

MSAT task data was checked for completeness, i.e. number of trials in which the participant did not select at least one moral emotion / action tendency. Moreover, participants were instructed to restrict their choice to only one moral emotion / action tendency. However, some participants selected more than one choice and such MSAT task trials were excluded from the analysis. Participants with less than 80% valid trials were excluded from the overall analysis (MSAT: n = 5/39 MDD, 1/14 control).

Means and standard deviations were computed for the proportion of moral emotions / action tendencies chosen in each condition (self- and other-agency). In addition, blame attribution and self-contempt bias scores were derived. Blame attribution was calculated for each participant by subtracting the average other-blame ratings from the average self-blame ratings in the self-agency condition (i.e. agency-congruent self-blaming bias) and in the other-agency condition (i.e. agency-incongruent self-blaming bias). Self-contempt bias scores were calculated by subtracting the percentage of other-contempt/disgust in the other-agency condition from the percentage of self-contempt/disgust in the self-agency condition, as described previously (Green et al., 2013; Zahn et al., 2015).

# Supplementary Results

## *Sequence optimisation: comparison TRF vs short TE*

Overall, both the short TE sequence and the TRF sequence (first pilot and second pilot) had good signal quality, with most regions achieving the minimum threshold of 40 as proposed by Murphy, Bodurka, and Bandettini (2007) (Supplementary Figure 1). However, the short TE had a better tSNR compared to both pilots using the TRF sequence with regard to the main ROIs (Supplementary Table 1). As qualitative feedback indicated that the TRF sequence caused the monitor screen to vibrate, which made it hard to read the statements, the short TE sequence was selected for the study.

## *fMRI findings*

When categorising the participants into partial responders (i.e. participants who showed at least a 25% reduction in depressive symptoms as measured on the QIDS-SR16) and non-responders, partial responders displayed higher RSATL – posterior subgenual cortex (BA25) connectivity during self-blame and lower RSATL – posterior subgenual cortex (BA25) connectivity during other-blame, while non-responders showed the opposite pattern (Supplementary Figure 2). Using the *a priori* posterior subgenual cortex (BA25) ROI to extract the PPI effect, these RSATL – posterior subgenual cortex (BA25) connectivity differences were driven by an interaction between condition (self- vs other-blaming) and group (partial responders vs non-responders; *F*(1,32) = 6.95, *p* = .013). There was no main effect of condition (*F*(1,32) = .11, *p* = .74) or group (*F*(1,32) = 1.95, *p* = .17). The interaction effect was driven by partial responders showing higher RSATL – posterior subgenual cortex (BA25) connectivity during self-blame (M = .15, SD = .79) relative to other-blame (M = -.47, SD = .58), resulting in a positive difference for self- vs other-blame (M = .62, SE = .33, *t* = 1.86, df = 14, *p* = .08), whereas non-responders showed lower RSATL – posterior subgenual cortex (BA25) connectivity during self-blame (M = -.20, SD = .82) relative to other-blame (M = .28, SD = .71), resulting in a negative difference for self- vs other-blame (M = -.48, SE = .26, *t* = -1.84, df = 18, *p* = .08). There was a significant difference between groups on self-blame vs other-blame differences (mean difference = 1.10, SE = .42, *t* = 2.64, df = 32, *p* = .013), which was identified by the observed interaction effect. When including the reserve list, the interaction effect remained (*F*(1,37) = 5.45, *p* = .03), indicating that it was unlikely to be driven by selecting a subset of participants.

## *Exploratory cross-sectional fMRI findings*

Using a two-way factorial SPM model probing group (MDD vs control) and condition (self- vs other-blaming) effects on BOLD activation, small-volume correction with our pre-registered Tier 1 ROIs (i.e. posterior subgenual cortex (BA25) and right striatum / pallidum) did not uncover any main effect of group or condition or an interaction effect. In contrast, small-volume correction with our Tier 2 ROIs identified an interaction effect between condition (self- vs other-blaming) and group (MDD vs control) for the right anterior subgenual cingulate (BA24/BA32; *F* = 9.96, voxel-based FWE-corrected *p* = .047), but no effects for the frontopolar cortex (BA10) (Supplementary Table 2).

As illustrated by Supplementary Figure 3 (whole-brain derived cluster averages), this interaction effect was driven by the control group showing lower anterior subgenual cingulate (BA24/BA32) signal for other-blame (M = -1.43, SD = 1.71) relative to self-blame (M = .24, SD = 2.49), resulting in a positive difference for self- vs other-blame (M = 1.69, SE = .67). In contrast, there was no anterior subgenual cingulate (BA24/BA32) signal difference in the MDD group between other-blame (M = -.30, SD = 1.67) relative to self-blame (M = -.38, SD = 1.59; difference for self-blame vs other-blame: M = -.09, SE = .27). Consequently, the groups differed on self-blame vs other-blame differences (mean difference = -1.75, SE = .59), which was identified by the observed interaction effect.

In terms of fMRI connectivity as examined by PPI analysis, small-volume correction with either Tier 1 or Tier 2 ROIs did not show any main effect of group or condition, nor an interaction effect.

## *MSAT findings*

Most statements were able to evoke moderate to strong feelings of self- and other-blame (Supplementary Table 5). MDD participants and controls attributed similar levels of blame to themselves (5.3 vs 4.8, respectively; *t*(58) = 1.87, *p* = .07) and to their friend (1.9 vs 1.6, respectively; *t*(58) = 1.08, *p* = .28) in the self-blame agency. Interestingly, MDD participants attributed more blame to themselves in the other-blame agency relative to controls (2.9 vs 1.7, respectively; *U*(60) = 182.0, *z* = -3.30, *p* = .001), while attributing similar levels of blame to their friends in the other-blame agency (4.0 vs 4.4, respectively; *t*(58) = -1.06, *p* = .29). As a result, MDD participants displayed more agency-incongruent self-blaming bias relative to controls (-1.1 vs -2.6, respectively; *U*(60) = 203.0, *z* = -2.97, *p* = .003), while the groups displayed similar levels of agency-congruent self-blaming bias (3.5 vs 3.1, respectively; *t*(58) = 1.02, *p* = .31).

In addition, self-contempt biases were increased in the MDD group compared to the control group (13.4 vs -1.5, respectively; *U*(60) = 159.5, *z* = -3.67, *p* < .001).

**Supplementary Figure Legends**

**Supplementary Figure 1 | Pilot data of one volunteer used to determine temporal signal-to-noise ratios (tSNRs).**

There were three separate sessions of the optimised value-related moral sentiment task, either using a tailored radio frequency (TRF) sequence (twice) or a short time-echo (TE) sequence. Overall, the short TE sequence had a better tSNR compared to the TRF sequence (session 1 and 2). However, both sequences exceeded the minimum threshold of 40 for most regions as proposed by Murphy et al. (2007). Panel A shows a sagittal view, Panel B shows an axial view, and Panel C shows a coronal view. The colour bar represents tSNR values. Displayed using MRIcron (Rorden & Brett, 2000).

**Supplementary Figure 2 | Sagittal view of connectivity between right superior anterior temporal lobe seed region and posterior subgenual cortex (BA25) for self-blaming vs other-blaming emotions.** The panel shows sagittal slices of the significant posterior subgenual cortex (BA25) cluster, displayed using MRIcron (Rorden & Brett, 2000) at an uncorrected voxel-level threshold of *p* = .005, with no cluster-size threshold (the colour bar represents *t* values; the numbers below the brain slices stand for x-coordinates of the Montreal Neurological Institute coordinate system). BA = Brodmann Area.

**Supplementary Figure 3 | Interaction effect between group (partial responders vs non-responders) and condition (self- vs other-blaming) for RSATL – posterior subgenual cortex (BA25) connectivity**

There was an interaction effect between group (partial responders vs non-responders, where partial responder was defined as participants who showed at least a 25% reduction in depressive symptoms as measured on the QIDS-SR16) and condition (self- vs other-blaming) for RSATL – posterior subgenual cortex (BA25) connectivity, using the extracted *a priori* posterior subgenual cortex (BA25) ROI averages. The interaction effect was driven by higher RSATL – posterior subgenual cortex (BA25) connectivity during self-blame in the partial response group compared to the non-response group, and lower RSATL – posterior subgenual cortex (BA25) connectivity during other-blame in the partial response group compared to the non-response group. There was a difference between groups on self-blame vs other-blame differences, which was identified by the observed interaction effect. RSATL = right superior anterior temporal lobe; BA = Brodmann Area; QIDS-SR16 = Quick Inventory of Depressive Symptomatology – self-rated, 16 items; ROI = region-of-interest.

**Supplementary Figure 4 |** **BOLD interaction effect between group (MDD vs control) and condition (self- vs other-blaming) in the right anterior subgenual cingulate cortex (BA24/BA32).**

A cropped section through the right anterior subgenual cingulate cortex (BA24/BA32), displayed using MRIcron (Rorden & Brett, 2000) at an uncorrected voxel-level threshold of *p* = .005, with no cluster-size threshold (the colour bar represents *F* values; the number above the brain slice stand for the x-coordinate of the Montreal Neurological Institute coordinate system). This interaction was due to lower anterior subgenual cingulate (BA24/BA32) signal for other-blame in the control group compared to the MDD group, and higher anterior subgenual cingulate (BA24/BA32) signal for self-blame in the control group compared to the MDD group. There was a main effect of condition, but no main effect of group. Anterior subgenual cingulate (BA24/BA32) regression coefficients were derived from the whole-brain analysis cluster. MDD = major depressive disorder; BOLD = blood-oxygen level-dependent; BA = Brodmann Area.

## Supplementary Tables

**Supplementary Table 1 | Overview of inclusion / exclusion for imaging analysis.**

|  | MDD | Control | *Total* |
| --- | --- | --- | --- |
| Total: | 45 | 20 | *65* |
| Included in main analysis: | 34 | 13 | *47* |
| - Reserve list, applying less stringent movement criteria (translation < 8 mm; rotation < 6 degrees) | 5 | 2 | *7* |
| Excluded: | 6 | 5 | *11* |
| - Excluded – abnormal images with functional implications | 0 | 1 | *1* |
| - Excluded – excessive movement, but OK coverage | 2 | 1 | *3* |
| - Excluded – excessive dropout, but OK movement | 3 | 3 | *6* |
| - Excluded – excessive dropout and movement | 1 | 0 | *1* |
| MDD = major depressive disorder. | | |  |

**Supplementary Table 2 | Comparison tSNR values of short TE and TRF sequences for hard-to-image ROIs relevant to moral and emotional cognition.**

|  | RSATL | Anterior subgenual cingulate cortex (BA24) | Posterior subgenual cortex (BA25) |
| --- | --- | --- | --- |
| *Short TE* | 188.9 | 141.3 | 151.1 |
| *TRF - first pilot* | 81.2 | 84.9 | 111.6 |
| *TRF - second pilot* | 74.6 | 99.2 | 115.6 |
| tSNR = temporal signal-to-noise ratio; TE = echo time; TRF = tailored radio frequency; ROIs = regions-of-interest; RSATL = right superior anterior temporal lobe; BA = Brodmann Area. | | | |

**Supplementary Table 3 | Baseline demographic characteristics by group.**

|  | MDD | Control | Comparison |
| --- | --- | --- | --- |
|  | n = 39 | n = 15 |  |
| Age | 42.2 ± 14.8; 19 - 66 | 40.1 ± 12.5; 20 - 66 | *t*(52) = .48, *p* = .63 |
| Gender |  |  | χ^2^ (2,54) = .44, *p* = .80 |
| Female | n = 32 (82%) | n = 13 (87%) |  |
| Male | n = 6 (15%) | n = 2 (13%) |  |
| Other | n = 1 (3%) | n = 0 (0%) |  |
| Ethnicity^a^ |  |  | χ^2^ (1,53) = 3.72, *p* = .05 |
| Asian | n = 4 (10%) | n = 0 (0%) |  |
| Black | n = 2 (1%) | n = 0 (0%) |  |
| Other | n = 2 (1%) | n = 0 (0%) |  |
| White | n = 30 (77%) | n = 15 (100%) |  |
| Native first language |  |  | χ^2^ (1,54) = .97, *p* = .32 |
| English | n = 31 (80%) | n = 10 (67%) |  |
| Non-English | n = 8 (21%) | n = 5 (33%) |  |
| Years of education | 16.9 ± 3.6; 10 - 24 | 16.2 ± 3.1; 9 - 22 | *t*(52) = .62, *p* = .54 |
| ^a^ Missing data for one MDD; categories have been merged into White vs non-White for chi-square test.  Means, standard deviations and range are reported (*M ± SD; minimum – maximum).* Percentages may not add up to 100 due to rounding. * significant at *p* < .05, two-tailed. MDD = major depressive disorder. | | | |

**Supplementary Table 4 | Movement parameters, unpleasantness ratings and response times for self- and other-blaming emotion trials by group.**

|  | MDD | Control | Comparison |
| --- | --- | --- | --- |
|  | n = 39 | n = 15 |  |
| Movement parameters |  |  |  |
| RMS translation | .08 ± .04 | .10 ± .07 | *t*(16.8) = -.91, *p* = .37 |
| RMS rotation | .08 ± .04 | .09 ± .08 | *t*(52) = -.58, *p* = .57 |
| Unpleasantness^a^ (%) |  |  |  |
| Self-blaming emotion | 42.3 ± 19.2 | 43.2 ± 20.1 | *t*(51) = -.15, *p* = .88 |
| Other-blaming emotion | 57.4 ± 18.9 | 40.7 ± 16.2 | *t*(51) = 3.01, *p* = .004* |
| Response time^a^ (ms) |  |  |  |
| Self-blaming emotion | 2162 ± 529 | 2149 ± 482 | *t*(51) = .08, *p* = .94 |
| Other-blaming emotion | 2227 ± 549 | 2372 ± 572 | *t*(51) = -.86, *p* = .39 |
| ^a^ One MDD participant had a faulty button box, so no behavioural measures were recoded.  Unpleasantness based on percentage of “quite unpleasant”. Means and standard deviations are reported (*M ± SD).* * significant at *p < .*05 threshold, two-tailed. MDD = major depressive disorder; RMS = root mean square. | | | |

**Supplementary Table 5 | Emotional relevance of MSAT stimuli as captured by blame-ratings and self-contempt bias.**

|  | MDD | Control | Comparison |
| --- | --- | --- | --- |
|  | n = 41 | n = 19 |  |
| Self-blame agency |  |  |  |
| Self-blame rating | 5.3 ± 1.1 | 4.8 ± 1.1 | *t*(58) = 1.87, *p* = .07 |
| Other-blame rating | 1.9 ± .8 | 1.6 ± .7 | *t*(58) = 1.08, *p* = .28 |
| Other-blame agency |  |  |  |
| Self-blame rating | 2.9 ± 1.5 | 1.7 ± .7 | *U*(60) = 182.0, *z* = -3.30, *p* = .001* |
| Other-blame rating | 4.0 ± 1.1 | 4.4 ± 1.3 | *t*(58) = -1.06, *p* = .29 |
| Self-blaming bias^a^ |  |  |  |
| Agency-congruent | 3.5 ± 1.2 | 3.1 ± 1.3 | *t*(58) = 1.02, *p* = .31 |
| Agency-incongruent | -1.1 ± 1.9 | -2.6 ± 1.3 | *U*(60) = 203.0, *z* = -2.97, *p* = .003* |
| Self-contempt bias^b^ | 13.4 ± 19.0 | -1.5 ± 8.2 | *U*(60) = 159.5, *z* = -3.67, *p* < .001** |
| ^a^ Self-blaming bias was calculated for each participant by subtracting average other-blame ratings from average self-blame ratings in each condition (self-agency [congruent] and other-agency [incongruent]).  ^b^ Self-contempt bias was computed for each participant by subtracting the percentage of other-contempt/disgust in the other-agency condition from the percentage of self-contempt/disgust in the self-agency condition.  Means and standard deviations are reported (*M ± SD).* * significant at *p < .*05 threshold, two-tailed. MDD = major depressive disorder; MSAT = moral sentiment and action tendencies. | | | |

**Supplementary Table 6 | Baseline clinical characteristics control participants (n=15).**

| Past depressive symptoms not meeting MDE criteria | 4 (27%) |
| --- | --- |
| Life-time axis-I disorder using DSM-5 criteria |  |
| Anxiety disorder | 5 (33%) |
| Subthreshold past posttraumatic stress disorder | 2 (13%) |
| None | 9 (60%) |
| Family history |  |
| First degree relative with probable MDD | 2 (13%) |
| No family history of MDD | 13 (87%) |
| MDD = major depressive disorder; MDE = major depressive episode; DSM-5 = Diagnostic and Statistical Manual for Mental Disorders 5^th^ edition. | |

**Supplementary Table 7 | Current and past MDD treatment (n=39).**

| Treatment at baseline |  |
| --- | --- |
| SSRI | 32 (82%) |
| *Sertraline* | 11 (28%) |
| *Citalopram* | 8 (21%) |
| *Escitalopram* | 3 (8%) |
| *Fluoxetine* | 5 (13%) |
| *Venlafaxine (≤ 150mg)* | 5 (13%) |
| SNRI | 4 (10%) |
| *Duloxetine* | 2 (5%) |
| *Venlafaxine (> 150mg)* | 2 (5%) |
| Tricyclic antidepressant | 2 (5%) |
| Other antidepressant | 1 (3%) |
| Add-on treatment | 4 (10%) |
| Non-pharmacological treatment | 10 (26%) |
| Past treatment |  |
| 1 – 2 medications | 26 (67%) |
| 3 – 4 medications | 9 (23%) |
| 5 – 6 medications | 4 (10%) |
| SSRI |  |
| *Sertraline* | 11 (28%) |
| *Citalopram* | 20 (51%) |
| *Escitalopram* | 4 (10%) |
| *Fluoxetine* | 22 (56%) |
| *Paroxetine* | 4 (10%) |
| *Venlafaxine (≤ 150mg)* | 5 (13%) |
| SNRI |  |
| *Duloxetine* | 2 (5%) |
| *Venlafaxine (> 150mg)* | 1 (3%) |
| Tricyclic antidepressant | 4 (10%) |
| Other antidepressant | 8 (21%) |
| Add-on treatment | 6 (15%) |
| Lifetime mental health/psychotherapy service use | 38 (97%) |
| *Of which past secondary care use* | 9 (23%) |
| Percentages may not add up to 100 due to rounding. MDD = major depressive disorder; SSRI = selective serotonin reuptake inhibitor; SNRI = selective norepinephrine reuptake inhibitor | |

**Supplementary Table 8 | MDD treatment during follow-up period (n=39).**

| Main change |  |
| --- | --- |
| No change in antidepressant | 21 (54%) |
| Stopped antidepressant | 6 (15%) |
| Lowered dose of antidepressant | 0 (0%) |
| Increase from effective dose to higher effective dose | 6 (15%) |
| Increase from ineffective dose to effective dose | 0 (0%) |
| Change to another antidepressant at effective dose | 4 (10%) |
| Change to another antidepressant at ineffective dose | 2 (5%) |
| Main antidepressant |  |
| SSRI | 26 (67%) |
| *Sertraline* | 8 (21%) |
| *Citalopram* | 6 (15%) |
| *Escitalopram* | 4 (10%) |
| *Fluoxetine* | 3 (8%) |
| *Venlafaxine (≤ 150mg)* | 6 (15%) |
| SNRI | 4 (10%) |
| *Duloxetine* | 2 (5%) |
| *Venlafaxine (> 150mg)* | 2 (5%) |
| Mirtazapine | 3 (8%) |
| Tricyclic antidepressant | 1 (3%) |
| Other antidepressant | 0 (0%) |
| Add-on treatment | 5 (13%) |
| Change in mental health service use |  |
| Started accessing mental health service | 7 (18%) |
| Continued care in mental health service | 9 (23%) |
| Stopped mental health treatment | 3 (8%) |
| Type of mental health service use |  |
| *CBT* | 3 (8%) |
| *Psychotherapy* | 5 (13%) |
| *Psychoanalysis* | 1 (3%) |
| *Counselling* | 2 (5%) |
| *Other* | 5 (13%) |
| Percentages may not add up to 100 due to rounding. MDD = major depressive disorder; SSRI = selective serotonin reuptake inhibitor; SNRI = selective norepinephrine reuptake inhibitor; CBT = cognitive behavioural therapy. | |

**Supplementary Table 9 | Association between potential clinical confounders and percentage change for primary analysis MDD group (n=34).**

|  |  | QIDS-SR16 percentage change |
| --- | --- | --- |
| MM-PHQ-9 (baseline) | rho | .202 |
|  | *p*-value | .25 |
| GAD-7 (baseline) | rho | .172 |
|  | *p*-value | .33 |
| Current MDE duration (months) | rho | .420^*^ |
|  | *p*-value | .01 |
| Age of onset first MDE (years) | rho | -.168 |
|  | *p*-value | .34 |
| Number of MDE in lifetime | rho | -.250 |
|  | *p*-value | .15 |
| Total duration depression from onset (years) | rho | -.004 |
|  | *p­*-value | .98 |
| Number of suicide attempts | rho | .141 |
|  | *p­*-value | .43 |
| * significant at *p* < .05 threshold, two-tailed. MDD = major depressive disorder; QIDS-SR16 = Quick Inventory of Depressive Symptomatology – self-rated, 16 items; MM-PHQ-9 = Maudsley Modified Patient Health Questionnaire, 9 items; GAD-7 = Generalized Anxiety Disorder, 7 items; MDE = Major Depressive Episode. | | |

**Supplementary Table 10 | Two-way factorial model for fMRI activation (BOLD) in participants with MDD and controls (n = 47).**

|  |  |  |  | *MNI peak coordinates* | | |  |  |
| --- | --- | --- | --- | --- | --- | --- | --- | --- |
| Hemi-sphere | Region | Cluster size | Brodmann Area | x | y | z | F-value | Voxel-based FWE-corrected *p* value |
| BOLD model - group x condition interaction effect: | | | | | | | | |
| right | Anterior subgenual cingulate cortex | 27 | 24/32 | 3 | 29 | -10 | 9.96 | .047^a^ |
| ^a^ Using *a priori* bilateral subgenual cingulate region of interest (6 mm sphere, MNI: x = -4 / +4, y = 23, z = -5, Green et al. (2012)) for multiple comparison correction. There were no main effects of group or agency in this region. No voxels survived voxel-based FWE-correction over the whole brain at *p* = .05 for main effects or interactions.  BOLD = blood-oxygen level-dependent; MDD = major depressive disorder; FWE = Family-Wise Error; MNI = Montreal Neurological Institute. | | | | | | | | |

# References

Bennett, C. M., & Miller, M. B. (2010). How reliable are the results from functional magnetic resonance imaging? *Ann N Y Acad Sci, 1191*, 133-155. doi:10.1111/j.1749-6632.2010.05446.x

Brett, M., Anton, J.-L., Valabregue, R., & Poline, J.-B. (2002). *Region of interest analysis using an SPM toolbox.* Paper presented at the 8th International Conference on Functional Mapping of the Human Brain, Sendai, Japan.

Duan, S., Lawrence, A. J., Valmaggia, L., Moll, J., & Zahn, R. (2022). Maladaptive blame-related action tendencies are associated with vulnerability to major depressive disorder. *J Psychiatr Res, 145*, 70-76. doi:10.1016/j.jpsychires.2021.11.043

Duan, S., Valmaggia, L., Fennema, D., Moll, J., & Zahn, R. (2023). Remote virtual reality assessment elucidates self-blame-related action tendencies in depression. *J Psychiatr Res, 161*, 77-83. doi:10.1016/j.jpsychires.2023.02.031

Fennema, D. (2022). *Neural signatures of emotional biases and prognosis in treatment-resistant depression.* (PhD). King's College London, London.

Fennema, D., O'Daly, O., Barker, G. J., Moll, J., & Zahn, R. (2021). Internal reliability of blame-related functional MRI measures in major depressive disorder. *Neuroimage Clin, 32*, 102901. doi:10.1016/j.nicl.2021.102901

First, M. B., Williams, J. B. W., Karg, R. S., & Spitzer, R. L. (2015). *Structured Clinical Interview for DSM-5 - Research Version (SCID-5 for DSM-5, Research Version; SCID-5-RV, Version 1.0.0)*. Arlington, VA: American Psychiatric Association.

Green, S., Lambon Ralph, M. A., Moll, J., Deakin, J. F., & Zahn, R. (2012). Guilt-selective functional disconnection of anterior temporal and subgenual cortices in major depressive disorder. *Arch Gen Psychiatry, 69*(10), 1014-1021. doi:10.1001/archgenpsychiatry.2012.135

Green, S., Moll, J., Deakin, J. F., Hulleman, J., & Zahn, R. (2013). Proneness to decreased negative emotions in major depressive disorder when blaming others rather than oneself. *Psychopathology, 46*(1), 34-44. doi:10.1159/000338632

Harrison, P., Carr, E., Goldsmith, K., Young, A. H., Ashworth, M., Fennema, D., . . . Zahn, R. (2020). Study protocol for the antidepressant advisor (ADeSS): a decision support system for antidepressant treatment for depression in UK primary care: a feasibility study. *BMJ Open, 10*(5), e035905. doi:10.1136/bmjopen-2019-035905

Harrison, P., Walton, S., Fennema, D., Duan, S., Jaeckle, T., Goldsmith, K., . . . Zahn, R. (2021). Development and validation of the Maudsley Modified Patient Health Questionnaire (MM-PHQ-9). *BJPsych Open, 7*(4), e123. doi:10.1192/bjo.2021.953

Jaeckle, T. (2018). *Neurocognitive basis and treatment of self-blaming emotional biases in major depressive disorder.* King's College London,

Lawrence, A. J., Stahl, D., Duan, S., Fennema, D., Jaeckle, T., Young, A. H., . . . Zahn, R. (2022). Neurocognitive measures of self-blame and risk prediction models of recurrence in major depressive disorder. *Biol Psychiatry Cogn Neurosci Neuroimaging, 7*(3), 256-264. doi:10.1016/j.bpsc.2021.06.010

Lythe, K. E., Gethin, J. A., Workman, C. I., Lambon Ralph, M. A., Deakin, J. F. W., Moll, J., & Zahn, R. (2020). Subgenual activation and the finger of blame: individual differences and depression vulnerability. *Psychol Med*, 1-9. doi:10.1017/S0033291720003372

Lythe, K. E., Moll, J., Gethin, J. A., Workman, C. I., Green, S., Lambon Ralph, M. A., . . . Zahn, R. (2015). Self-blame-selective hyperconnectivity between anterior temporal and subgenual cortices and prediction of recurrent depressive episodes. *JAMA Psychiatry, 72*(11), 1119-1126. doi:10.1001/jamapsychiatry.2015.1813

Moll, J., de Oliveira-Souza, R., Garrido, G. J., Bramati, I. E., Caparelli-Daquer, E. M., Paiva, M. L., . . . Grafman, J. (2007). The self as a moral agent: linking the neural bases of social agency and moral sensitivity. *Soc Neurosci, 2*(3-4), 336-352. doi:10.1080/17470910701392024

Moll, J., De Oliveira-Souza, R., & Zahn, R. (2008). The neural basis of moral cognition: sentiments, concepts, and values. *Ann N Y Acad Sci, 1124*, 161-180. doi:10.1196/annals.1440.005

Moll, J., Krueger, F., Zahn, R., Pardini, M., De Oliveira-Souza, R., & Grafman, J. (2006). Human fronto-mesolimbic networks guide decisions about charitable donation. *Proc Natl Acad Sci U S A, 103*(42), 15623-15628. doi:10.1073/pnas.0604475103

Montgomery, S. A., & Asberg, M. (1979). A new depression scale designed to be sensitive to change. *Br J Psychiatry, 134*, 382-389. doi:10.1192/bjp.134.4.382

Murphy, K., Bodurka, J., & Bandettini, P. A. (2007). How long to scan? The relationship between fMRI temporal signal to noise ratio and necessary scan duration. *Neuroimage, 34*(2), 565-574. doi:10.1016/j.neuroimage.2006.09.032

Ojemann, J. G., Akbudak, A., Snyder, A. Z., McKinstry, R. C., Raichle, M. E., & Conturo, T. E. (1997). Anatomic localization and quantitative analysis of gradient refocused echo-planar fMRI susceptibility artifacts. *Neuroimage, 6*(3), 156-167. doi:10.1006/nimg.1997.0289

Peirce, J., Gray, J. R., Simpson, S., MacAskill, M., Hochenberger, R., Sogo, H., . . . Lindelov, J. K. (2019). PsychoPy2: Experiments in behavior made easy. *Behav Res Methods, 51*(1), 195-203. doi:10.3758/s13428-018-01193-y

Rorden, C., & Brett, M. (2000). Stereotaxic display of brain lesions. *Behav Neurol, 12*(4), 191-200. doi:10.1155/2000/421719

Rush, A. J., Trivedi, M. H., Ibrahim, H. M., Carmody, T. J., Arnow, B., Klein, D. N., . . . Keller, M. B. (2003). The 16-item Quick Inventory of Depressive Symptomatology (QIDS), clinician rating (QIDS-C), and self-report (QIDS-SR): a psychometric evaluation in patients with chronic major depression. *Biol Psychiatry, 54*(5), 573-583. doi:10.1016/s0006-3223(02)01866-8

Savitz, J. B., Rauch, S. L., & Drevets, W. C. (2013). Clinical application of brain imaging for the diagnosis of mood disorders: the current state of play. *Mol Psychiatry, 18*(5), 528-539. doi:10.1038/mp.2013.25

Sheehan, D. V., Lecrubier, Y., Sheehan, K. H., Amorim, P., Janavs, J., Weiller, E., . . . Dunbar, G. C. (1998). The Mini-International Neuropsychiatric Interview (M.I.N.I.): the development and validation of a structured diagnostic psychiatric interview for DSM-IV and ICD-10. *J Clin Psychiatry, 59*, 22-33.

Spitzer, R. L., Kroenke, K., Williams, J. B. W., & Lowe, B. (2006). A brief measure for assessing generalised anxiety disorder: the GAD-7. *Arch Intern Med, 166*(10), 1092-1097. doi:10.1001/archinte.166.10.1092

Wastling, S. J., & Barker, G. J. (2015). Designing hyperbolic secant excitation pulses to reduce signal dropout in gradient-echo echo-planar imaging. *Magn Reson Med, 74*(3), 661-672. doi:10.1002/mrm.25444

Wood, J. N., & Grafman, J. (2003). Human prefrontal cortex: processing and representational perspectives. *Nat Rev Neurosci, 4*(2), 139-147. doi:10.1038/nrn1033

Zahn, R., de Oliveira-Souza, R., & Moll, J. (2020). Moral motivation and the basal forebrain. *Neurosci Biobehav Rev, 108*, 207-217. doi:10.1016/j.neubiorev.2019.10.022

Zahn, R., Green, S., Beaumont, H., Burns, A., Moll, J., Caine, D., . . . Lambon Ralph, M. A. (2017). Frontotemporal lobar degeneration and social behaviour: dissociation between the knowledge of its consequences and its conceptual meaning. *Cortex, 93*, 107-118. doi:10.1016/j.cortex.2017.05.009

Zahn, R., Lythe, K. E., Gethin, J. A., Green, S., Deakin, J. F., Workman, C., & Moll, J. (2015). Negative emotions towards others are diminished in remitted major depression. *Eur Psychiatry, 30*(4), 448-453. doi:10.1016/j.eurpsy.2015.02.005
